# Caffeic Acid Phenethyl Ester (CAPE) Inhibits Arginase Activity and Growth of *Leishmania amazonensis* Promastigotes and Intracellular Amastigotes

**DOI:** 10.3390/pathogens14040384

**Published:** 2025-04-15

**Authors:** Edson Roberto da Silva, André Mesquita, Claudia do Carmo Maquiaveli

**Affiliations:** Faculdade de Zootecnia e Engenharia de Alimentos, Universidade de São Paulo, Av. Duque de Caxias Norte, 225, Pirassununga 13635-900, SP, Brazil; andremqt@usp.br

**Keywords:** antileishamanials, arginase, caffeic acid, polyamines, cutaneous leishmaniasis

## Abstract

Caffeic acid phenethyl ester (CAPE) is a polyphenol produced by many plants and is found in red and green propolis. Here, we evaluated the antileishmanial activity of this natural product against *Leishmania amazonensis*. CAPE exhibited IC_50_ values of 8.07 µM (95% CI, 6.79–9.62 µM) and 13.51 µM (95% CI, 10.71–17.16 µM) against *L. amazonensis* promastigotes and intracellular amastigotes, respectively. Additionally, CAPE inhibited *L. amazonensis* arginase in a non-competitive manner with a K_i_ value of 1.51 ± 0.04 µM. These results highlight the potential of CAPE as a promising lead compound for developing new therapies against leishmaniasis.

## 1. Introduction

Leishmaniasis is a neglected tropical disease caused by protozoan parasites of the genus *Leishmania*. The parasites from the genus *Leishmania* are transmitted to humans and animals by a phlebotomine sandfly vector [1]. In the vector, *Leishmania* is present in the promastigotes form and inhabits the midgut of the insect. In animals, including humans, promastigotes are phagocytized by innate defense cells and promastigotes change to the amastigote form living inside phagocyte vacuoles [2].

The World Health Organization estimates that more than 1 billion humans live in endemic areas. Approximately 30,000 new cases of visceral leishmaniasis (VL) and over one million new cases of cutaneous leishmaniasis (CL) occur annually. CL occurs primarily in impoverished and developing countries worldwide, with an estimated one billion people living in leishmaniasis-endemic areas and who are at risk of infection [1]. The major challenges associated with these diseases include parasite resistance to currently available drugs, such as pentavalent antimonials, and the lack of new drug development [1]. Despite these challenges, using natural products or adequate nutrition may be valuable tools in managing this disease and preventing its progression [3].

*Leishmania* parasites can survive within host cells by producing trypanothione, an antioxidant that neutralizes the effects of nitric oxide (NO) on the parasite [4,5]. The first step in trypanothione biosynthesis involves the enzyme arginase, which initiates the polyamine pathway in *Leishmania*. Arginase hydrolyzes L-arginine to urea and ornithine, which is then decarboxylated by ornithine decarboxylase to produce putrescine, subsequently used to synthesize spermidine [4]. Finally, two molecules of glutathione and one molecule of spermidine are used to synthesize trypanothione by trypanothione synthetase [6]. Targeting arginase is considered a promising strategy for drug development because this enzyme plays a central role in *Leishmania* survival and pathogenesis [5,7,8].

Synthetic compounds derived from caffeic acid, initially synthesized to inhibit mammalian arginase as a potential therapeutic target for vascular disease [9], have shown specific arginase inhibition from *L. amazonensis* [10]. In contrast, arginase inhibitors designed for mammalian enzymes also inhibit *Leishmania mexicana* arginase [11].

**Figure 1:**
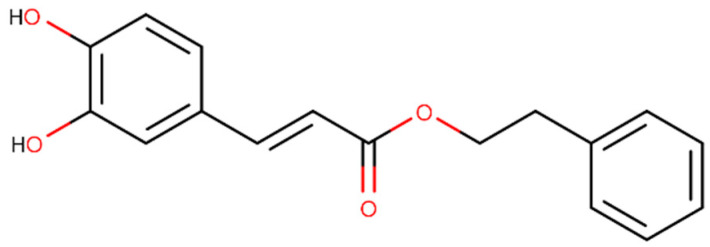
Chemical structure of caffeic acid phenethyl ester (CAPE), a natural compound found in propolis.

Caffeic acid phenethyl ester (CAPE, Figure 1) has demonstrated a range of biological activities beneficial to human and animal health, including kidney protection against free radicals in a carbon tetrachloride-induced animal model [12] and the induction of apoptosis in human leukemic HL-60 cells [13].

CAPE is a polyphenol synthesized by plants and is a major component of propolis produced in temperate regions. It has shown great potential in various therapeutic applications, including leishmaniasis [14,15,16,17,18]. This compound possesses numerous biological properties, including anti-inflammatory, antimicrobial, antioxidant, immunomodulatory, antiprotozoal, and antimitotic activities [15].

This study investigated the antileishmanial activity of CAPE against *Leishmania amazonensis* promastigotes and intracellular amastigotes, as well as its ability to inhibit arginase.

## 2. Materials and Methods

### 2.1. Materials

Caffeic acid phenethyl ester (CAPE), 3(4,5-dimethylthiazol-2y-l)-2,5 diphenyl bromide tetrazolium (MTT), thioglycolate, hemin, and fetal bovine serum were obtained from Sigma Aldrich, St. Louis, MO, USA. M-199 and RPMI 1640 culture media and penicillin/streptomycin were obtained from Life Technologie Corporation, Frederick, MD, USA.

### 2.2. Kinetics of Arginase Inhibition

An arginase assay was performed using purified recombinant arginase, as previously described [19]. Briefly, the IC_50_ was determined in a buffer containing CHES 50 mM and L-arginine 50 mM in pH 9.5. A 50 mM stock solution of CAPE was prepared in DMSO, followed by a dilution to 1 mM in Chess 50 mM. This 1 mM solution was then serially diluted using a 10-fold dilution factor to obtain CAPE concentrations ranging from 0.01 to 100 μM for the inhibition assay. The mechanism of enzyme inhibition was determined using three different concentrations of substrate (25, 50, and 100 mM) and three different concentrations of CAPE (2, 4, and 8 μM). The kinetic data were used to calculate Ki and Kis using a plot model described by Dixon and Cornish-Bowden and then to visualize the mechanism of enzyme inhibition graphically [20,21].

### 2.3. Promastigote Test Culture

Promastigotes of *L. amazonensis* (MHO/BR/1973/M2269) were grown in a M-199 culture medium supplemented with 10% of bovine serum, 100 U/mL of penicillin, 50 μg/mL of streptomycin, and 5 ppm hemin, and was maintained at 25 °C until the cells reached the stationary phase. Promastigotes (5.0 × 10^5^ cells/mL) were incubated with CAPE (dissolved in DMSO) at a final concentration between 1.5625–100 μM. The final DMSO concentration in the culture media was 0.2% and did not interfere with parasite growth. Amphotericin B was used as a positive control in a range from 10 to 0.001 µM. Tests were performed in a final volume of 1.0 mL using conical microtubes of 1.5 mL. After 72 h of incubation, the surviving cells were quantified using the MTT. Formazan quantification produced by the surviving cells was performed in the spectrophotometer. A total of two independent assays were performed in triplicate. The CAPE effect was expressed as IC_50_, which corresponds to the concentration that kills 50% of parasites [22].

### 2.4. Amastigote Culture

A total of 10 male Swiss murine mice (10–12 g) were used in this study. The animals were maintained in four per cage at a temperature of 22 °C with a light/dark cycle of 12 h and fed a standard diet *ad libitum*. The experiments were performed following the ethical principles for animal experimentation adopted by the Brazilian College of Animal Experimentation, and the Animal Experimentation Committee of the Faculty of Animal Science and Engineering of Food of the University of São Paulo (FZEA-USP) approved the study’s protocol (CEUA code 3086190918). The production of peritoneal macrophage was stimulated by the peritoneal administration of thioglycolate 3% (0.5 mL). On the following day of the thioglycolate administration, the animals were euthanized through cervical dislocation. After this procedure, the peritoneums of the animals were washed with 10 mL of PBS (phosphate buffer saline), and the peritoneal macrophages were collected with a sterile syringe and needle. The peritoneal macrophages were centrifuged at 10,000 rpm and transferred to the RPMI 1640 culture medium, which was supplemented with 10% of fetal bovine serum, 10 U/mL of penicillin, and 10 μg/mL of streptomycin. To determine the peritoneal macrophage infection index, 10^5^ cells were seeded into an 8-well glass chamber slide (Lab-Tek Chamber Slide, Nunc, Frederick, MD, USA) and incubated for 4 h at 34 °C in 5% CO_2_ [23].

Stationary phase promastigotes were added to the wells at a parasite-to-macrophage ratio of 10 to 1. After 6 h of incubation, the non-phagocytized promastigotes were washed away with a fresh medium. The infected macrophages were exposed to 0.2% DMSO diluted in the RPMI 1640 medium and serial dilution (1:2) CAPE, which was initially diluted in DMSO and subsequently in the RPMI medium to yield a final concentration that ranged from 50 to 3.125 μM. For the positive control of parasite growth inhibition, the standard drug Amphotericin B was diluted in DMSO and then in RPMI to reach a final concentration of 2 μM. After 72 h of the treatment, the infected macrophages were washed with fresh medium, and the slides were fixed with 100% methanol and stained with Giemsa. The infective index (the rate of infected macrophages multiplied by the average number of amastigotes per macrophage) was calculated by post-test, randomly counting at least 200 macrophages in the test slide. The CAPE effect was expressed with IC_50_, corresponding to a concentration that reduces 50% of the infective index compared with the control group. All experiments were performed in duplicate and repeated in three independent experiments.

### 2.5. Statistical Analysis

The IC_50_ for arginase inhibition was calculated using a four-parameter model, while the IC_50_ for promastigotes and amastigotes was calculated using a log inhibitor–response curve with a variable slope. The data were analyzed using GraphPad Prism software (version 10.1.2 (324), San Diego, CA, USA).

## 3. Results and Discussion

**Figure 2:**
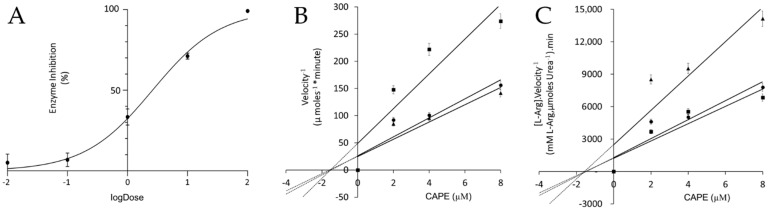
The arginase inhibition and mechanism of enzyme inhibition. (**A**) Dose–response curve for CAPE against *L. amazonensis* arginase. Dixon (**B**) and Cornish-Bowden (**C**) plots were used to determine the values of the constants Ki and Kis, respectively. Graphically, it is inferred that the *L. amazonensis* arginase inhibition mechanism is non-competitive (Ki = Kis). The concentrations of L-arginine used were 25 mM (■), 50 mM (●), and 100 mM (▲). Data represent the average of three experiments performed in duplicate. Error bars indicate the standard deviation of the mean from three independent experiments performed in duplicate. Data were fitted to a linear regression model (R^2^ = 0.85).

CAPE inhibited the *L. amazonensis* arginase with an IC_50_ value of 1.94 μM (95% CI of 1.44 to 2.63 µM, r^2^ = 0.95), demonstrating a non-competitive mechanism of inhibition with a K_i_ value of 1.51 ± 0.04 µM (Figure 2). In non-competitive inhibition, the inhibitor binds to the enzyme with equal affinity in both the presence and absence of the substrate L-arginine.

While the effect of CAPE on human arginase requires further investigation, the possibility of inhibiting this enzyme could offer a therapeutic advantage by promoting NO production, a crucial factor in macrophage-mediated parasite killing [24].

**Figure 3:**
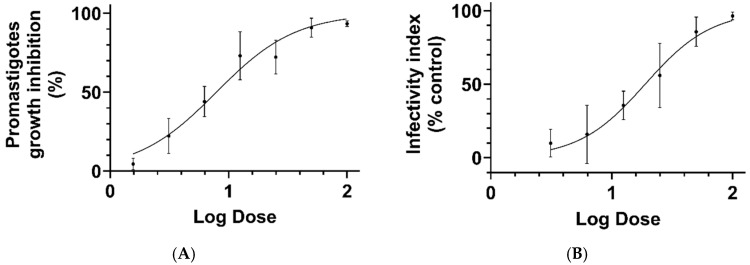
In vitro activity of CAPE against *L. amazonensis*. Dose–response curves showing the effect of CAPE on promastigotes growth (**A**) and infectivity index intracellular amastigotes (**B**). Data points represent the arithmetic mean of three independent experiments performed in duplicate. The viability of promastigotes treated with CAPE was quantified using the MTT assay. The infectivity index was calculated by direct counting of infected macrophages and the number of amastigotes inside parasitophorous vacuoles. At least 200 macrophages were observed to determine the percentage of infected cells and the number of amastigotes per infected macrophage.

The antileishmanial activity of CAPE was evaluated against promastigotes, and the compound exhibited an IC_50_ value of 8.07 µM (95% CI, 6.79–9.62 µM; R^2^ = 0.88). The IC_50_ in the amphotericin control group was 0.02 µM (0.01–0.03 µM, 95% CI, R^2^ = 0.95). CAPE was also effective against the intracellular amastigote form of the parasite, with an IC_50_ value of 13.51 µM (95% CI, 10.71–17.16 µM; R^2^ = 0.77) (Figure 3).

The toxicity of CAPE against J774 macrophage cells has been previously reported, with an IC_50_ of 42.0 ± 1.3 µg/mL [25].

Natural products are often used as a starting point for drug discovery and development. Caffeic acid-derived compounds have been extensively studied for their anti-inflammatory, antibacterial, and antiprotozoal properties [15]. Caffeic acid is a component of other naturally occurring bioactive-derived compounds, such as verbascoside, isoverbascoside [26], and rosmarinic acid [27]. Verbascoside shows an IC_50_ = 19 μM against promastigotes [22] and an IC_50_ of 32 μM against intracellular amastigotes [23], while rosmarinic acid shows an IC_50_ = 0.7 μM against promastigotes and an IC_50_ = 4.8 μM against the intracellular amastigotes of Verbascoside, isoverbascoside, and rosmarinic acid inhibits arginase from *L. amazonensis* [22,28].

Synthetic derivatives containing caffeic acid have been explored for the development of drugs for cardiovascular disease by targeting human arginase. Arginase and nitric oxide synthase (NOS) compete for the same substrate, L-arginine. NOS is a key enzyme that produces nitric oxide (NO) in the vascular endothelium [9]. During inflammation, macrophages express an inducible NOS to generate NO, which exhibits cytotoxic activity against microorganisms such as bacteria and *Leishmania* parasites [29]. Therefore, inhibiting *Leishmania* arginase is a potential therapeutic strategy for reducing L-arginine consumption by the parasite and increasing NO bioavailability for the host [5].

This study highlights the antileishmanial effects of CAPE and its potential link to arginase inhibition. The observed IC_50_ values against both promastigotes and intracellular amastigotes were in the micromolar range, indicating good potency of CAPE and its potential for drug development. The compound CAPA (caffeic acid phenethyl amide) was tested previously [10], but its IC_50_ value against promastigotes (80 µM) [10] was higher than that of CAPE (IC_50_ = 8 µM). While further investigation is needed, we speculate that the difference in potency between CAPE and CAPA against intracellular amastigotes may be related to their distinct molecular geometries. The square pyramidal geometry of the ester linkage in CAPE could be more conducive to interactions with target molecules, such as arginase or macrophage receptors, compared to the trigonal pyramidal geometry of the imine group in CAPA. The amide function may also be more susceptible to metabolic degradation.

The distinct modes of arginase inhibition exhibited by CAPA (competitive) and CAPE (non-competitive) offer a potential explanation for their differing activities against *L. amazonensis*. The parasite’s ability to modulate the L-arginine pool and increase substrate availability [30] could effectively counteract the competitive inhibition by CAPA. Conversely, CAPE’s non-competitive inhibition, independent of substrate concentration, remains effective even under conditions of elevated L-arginine levels. This physiologically relevant difference in inhibition mechanism could be a key factor in the observed differences in anti-parasitic activity. Furthermore, if CAPE inhibits human arginase, this could indirectly enhance leishmanicidal activity [24]. Reduced arginase activity would increase L-arginine availability for nitric oxide synthase, leading to increased nitric oxide production, a critical mechanism for killing Leishmania [31,32,33].

CAPE is a constituent of the ethanolic extract of Brazilian propolis, which was previously tested against *L. amazonensis* [16]. The antileishmanial effects of CAPE and nanoparticles loaded with it were evaluated against *L. infantum*, with promising results [25]. We characterized the effects of CAPE on the promastigotes and intracellular amastigotes of *L. amazonensis* and found IC_50_ values similar to those previously reported for *L. infantum*. Our results showed that the promastigotes of *L. amazonensis* were more sensitive to CAPE (IC_50_ = 8 µM or ~2.2 µg/mL) than those of *L. infantum* (IC_50_ = 19.0 µg/mL).

The potency difference between *L. amazonensis* and *L. infantum* was also observed with intracellular amastigotes. The IC_50_ against *L. amazonensis* was 3.8 µg/mL (13.5 µM), while the IC_50_ against *L. infantum* was 19 µg/mL, representing a roughly 5-fold difference [25].

The lower susceptibility of intracellular amastigotes compared to promastigotes is likely due to several factors. First, the drug must overcome additional barriers to reach its target. In the case of amastigotes residing within macrophages, CAPE needs to cross two extra membranes: the macrophage cell membrane and the phagosomal membrane. This can significantly reduce the effective concentration of the drug that reaches the target protein. Additionally, the metabolism of CAPE by esterase enzymes could contribute to the reduced efficacy against amastigotes. Hydrolysis of the ester bond in CAPE would inactivate the compound and release caffeic acid and 2-phenylethanol, which are inactive metabolites.

Furthermore, considering that the target of CAPE is arginase, a glycosomal *Leishmania* enzyme [7], the drug would need to cross the glycosomal membrane to exert its effect. This could pose an additional barrier and further reduce the accessibility of CAPE to its target, potentially contributing to the lower susceptibility of amastigotes. To fully understand the factors influencing CAPE’s activity against different *Leishmania* life cycle stages, further studies are needed to investigate its intracellular pharmacokinetics and metabolism, including its ability to penetrate glycosomes and reach arginase.

In this study, we utilized peritoneal macrophages (PEM) for the in vitro assessment of drug activity against *Leishmania* amastigotes. We observed promising activity for the natural compound CAPE. It is important to note that the results may vary in other host cell lineages. Seifert et al. [34] demonstrated that the potency of antileishmanial drugs can differ depending on the host cell type. For example, amphotericin B was more active in PEMs and BMMΦ compared with PBMΦ and differentiated THP-1 cells, while miltefosine was more active in PBMΦ compared with PEMs and BMMΦ. Sodium stibogluconate displayed the highest activity in PBMΦ.

In contrast to the variability observed between different host cell types, the surface markers of macrophages CD11b+, F4/80+, CD68+, and CD14+ were similar between the ex vivo cultured macrophages isolated from the mouse lesions induced by *L. amazonensis* and infected peritoneal macrophages [35].

The next steps in this research should include investigating the interaction of CAPE with human arginase and conducting in vivo studies to assess its antileishmanial activity and safety profile in a human host.

In conclusion, CAPE effectively inhibited the growth of *L. amazonensis* promastigotes and significantly reduced the infection index in an assay with intracellular amastigotes. Moreover, CAPE inhibited the arginase enzyme of the parasite.

## Data Availability

Data are available both within the article and upon request to E.R.d.S. as raw data.
